# Cholesterol inhibits capsaicin activation of the TRPV1 channel

**DOI:** 10.1080/19336950.2026.2630491

**Published:** 2026-02-16

**Authors:** Tal Brandwine-Shemmer, Nicolas A. Barbera, Irena Levitan, Baruch Minke

**Affiliations:** a Department of Medical Neurobiology Faculty of Medicine and Edmond and Lily Safra Center for Brain Sciences (ELSC), The Hebrew University, Jerusalem, Israel; b Division of Pulmonary, Critical Care, Sleep and Allergy, Department of Medicine, University of Illinois at Chicago, Chicago, IL, USA

**Keywords:** TRPV1 channel, capsaicin, cholesterol, methyl-β-cyclodextrin (MβCD), docking analysis, Gly563Ser TRPV1 mutation

## Abstract

TRPV1 is a polymodal ion channel activated by vanilloids, noxious heat, and pro-inflammatory signals. A recent cryo-EM structure of human TRPV1 bound to SAF312, a potent, selective, noncompetitive antagonist, revealed a cholesterol molecule occupying the vanilloid-binding pocket, a site well established as the activation locus for vanilloid agonists. This observation led us to test whether cholesterol functionally inhibits capsaicin-dependent TRPV1 activation. Using HEK293 cells heterologously expressing TRPV1, we found that membrane cholesterol enrichment markedly suppressed capsaicin-evoked currents at low agonist concentrations, whereas responses to saturating capsaicin were unaffected. The functional interaction between cholesterol and capsaicin was further supported by site-directed mutagenesis targeting the conserved Gly563, a residue within the S4-S5 linker of the vanilloid-binding pocket. The G563S mutation reduced the sensitivity to capsaicin and caused slow and incomplete deactivation; nevertheless, elevated cholesterol further suppressed capsaicin-evoked activity. Together, these findings support a model in which cholesterol competes with capsaicin at the vanilloid-binding pocket to inhibit activation of the TRPV1 channel.

## Introduction

TRPV1 is predominantly expressed in peripheral nociceptive neurons, where it mediates heat detection and pain signaling, but it is also found in several central and non-neuronal tissues. Although lipid modulation of TRPV1 activity is well documented, the underlying mechanisms remains incompletely understood [1,2]. Recently, the cryo-EM structure of human TRPV1 in complex with SAF312, a potent selective and noncompetitive antagonist, provided structural insights into its inhibitory mechanism together with advanced understanding of cholesterol regulation of TRPV1 activity [3]. SAF312 occupies the vanilloid-binding pocket [3,4], thereby preventing conformational rearrangements of the S4 and S5 helices of TRPV1 that are essential for channel gating [5]. Unexpectedly, a putative cholesterol molecule was observed to contribute to SAF312-mediated inhibition [3,6].

**Figure 1.**
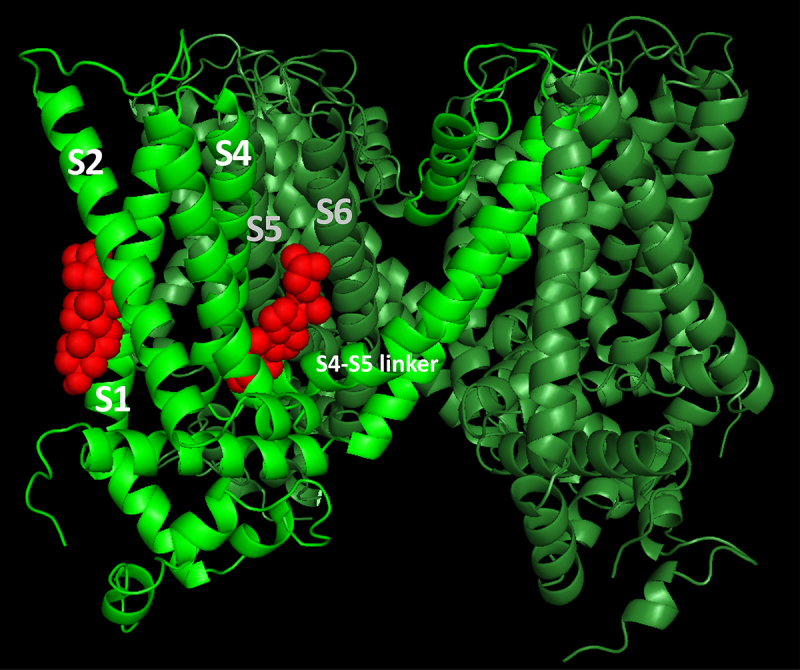
Predicted cholesterol-binding sites in the rTRPV1^WT^ channel using docking analysis. Two subunits of the rTRPV1 channel are shown, one in light green and the other in olive green. Cholesterol molecules (red) occupy two putative binding sites: (left) an annular site within a cleft between the S1 and S2 helices, and (right) a no-annular pocket located between two subunits, where the S5 and S6 helices of one subunit interact with the S4–S5 linker and the S4 helix of the adjacent subunit.

To further explore the functional implications of the interaction between TRPV1 and cholesterol, we performed molecular docking analysis combined with some functional tests. The docking algorithm generated multiple protein–ligand conformations and ranked them based on energetic stability, with the most favorable conformations representing the most probable binding modes. Examination of the 10 lowest-energy conformations revealed two predominant binding regions for cholesterol: one between the S1 and S2 segments at the protein–membrane interface, and another between the S5–S6 region of one subunit and S4 and S4–S5 linker of an adjacent subunit (Figure 1). The latter site is located deeper within the protein and exhibits minimal contact with the membrane. A similar binding pocket of cholesterol was identified by cryo-EM [3], located in the same region as the vanilloid-binding pocket [4,5]. Notably, the predicted binding of cholesterol in that region overlapped with a predicted binding of capsaicin (the hot ingredient of chili paper) [6]. This predicted co-localization of cholesterol and vanilloids within the vanilloid-binding pocket is consistent with the observed inhibition of capsaicin-evoked TRPV1 activation by cholesterol [7].

Molecular simulation-based experiments have shown that the distribution of the applied capsaicin is not uniform in the cell membrane and its highest concentration is at the lipid–water interface regions [7,8]. The importance of cholesterol in TRPV1 molecular function was demonstrated by Goswami and colleagues, who revealed that interaction between cholesterol and key Arg residues located at the cytosolic lipid–water interface is critical for TRPV1 stabilization and for conformational dynamics required for channel function [2]. Based on high-resolution cryo-EM structure of rat TRPV1, analysis of amino acid conservation in the lipid–water interface region and physio-chemical behavior of selected amino acids, they found that replacing Arg575 by Asp (R575D mutation) caused constitutive activity of the expressed TRPV1 and cell death, which could be rescued by chelation of extracellular Ca^2+^ or by reduction of membrane cholesterol [9]. Strikingly, Arg575 is localized in the S4–S5 linker, which constitutes an integral and important region of the vanilloid-binding pocket. In the present study, we directly tested whether cholesterol competes with capsaicin at the vanilloid-binding pocket to inhibit activation of the TRPV1 channel.

## Materials and methods

### Subcloning and mutagenesis

Rat TRPV1^WT^-mcherry was subcloned into pcDNA™4/TO mammalian expression vector. Site-directed mutagenesis was performed using PCR with primers harboring the desired mutation and a 5’ phosphate modification. The PCR products were extracted from agarose gel, purified, and ligated using the T4 ligase kit (Thermo Scientific) followed by transformation into DH5α competent cells. Insertion of mutations was confirmed by sequencing.

### Cell culture and transfection

T-REx™-293 cells were grown at 37°C in Dulbecco’s modified Eagle’s medium (DMEM) supplemented with 10% fetal bovine serum (Tet approved), 1% Pen-Strep, 2 mM l-glutamine (Biological Industries), 5 μg/mL Blasticidin (Invivogen). For transfections, cells were plated on poly-d-lysine-coated 16 mm glass coverslips 2 days prior to the experiments. The following day, cells were transiently transfected with either 1 μg of pCDNA4-rTRPV1^wt^-mcherry or pCDNA4-rTRPV1^G563S^-mcherry, along with 1 μg of pCDNA4-GFP. Transfections were performed using TransIT-LT1 (Mirus) transfection reagents, according to the manufacturer’s instructions and protocol. Expression of plasmids was induced with tetracycline (0.1 μg/mL).  

### Increasing cholesterol levels of the plasma membrane

A common method for modulating cholesterol levels in membranes involves methyl-β-cyclodextrin (MβCD), a cyclic oligomer of seven glucose units with high affinity for cholesterol [10]. In its empty form, MβCD is used to sequester cholesterol from membranes. In its cholesterol-loaded form (MβCD:cholesterol) it is used to enrich membranes with cholesterol.

For cholesterol enrichment of the plasma membrane, an MβCD:cholesterol complex was used, prepared as previously described [11]. Briefly, to prepare 100 mL of 5 mM MβCD:cholesterol (8:1), 50 mg of cholesterol was dissolved in 1 mL of chloroform:methanol (1:1). Then, 490 µL of dissolved cholesterol was transferred to a clean, open glass tube and left to evaporate. Separately, 655 mg of MβCD was dissolved into 100 mL of extracellular solution. Once fully dissolved, the MβCD solution was added to the dry cholesterol containing tube. The tube was then closed and vortexed until all dry cholesterol detached from the glass walls. The solution was then sonicated at maximal frequency for an additional 10–15 min. The solution was incubated overnight in a 37°C heated shaker. The next day, it was filtered and stored in room temperature for no longer than 6 days.

### Electrophysiology

For whole-cell recordings, borosilicate patch pipettes with a resistance of 2–4 MΩ were used. Currents were low-pass-filtered at 5 kHz and sampled at 10 kHz. A voltage ramp protocol ranging from −150 mV to +150 mV was applied. The extracellular solution contained (in mM): 147 NaCl, 5 KCl, 1 MgCl_2_, 1 HEPES, and 10 d-glucose, adjusted to pH 7.4 with NaOH. The pipette solution contained (in mM): 120 CsMeSO_3_, 25 CsCl, 1 MgCl_2_, 5 EGTA, 3 MgATP, 0.3 NaGTP, and 10 HEPES, adjusted to pH 7.4 with CsOH. Currents were recorded using an Axopatch 200B patch-clamp amplifier (Molecular Devices) and digitized via Digidata 1320A interface board (Axon instruments) with pCLAMP 10.6 software (Molecular Devices). All experiments were performed at room temperature.

Whole-cell patch clamp experiments were performed in T-REx™-293 cells transiently expressing rTRPV1^wt^, with expression induced 2.25–2.5 h prior to the experiments. This short induction period ensured low expression levels, aiming to prevent rTRPV1^wt^ constitutive currents. After establishing the whole-cell configuration and allowing basal currents to stabilize, the protocol began with capsaicin application to evoke TRPV1 currents, followed by a thorough washout with 10 mL of extracellular solution. Cells were then perfused with 5 mM MβCD:cholesterol (8:1) solution (4 mL, sufficient to fill the ~2 mL bath volume). This was followed by a 10-min incubation period, during which continuous perfusion of MβCD:cholesterol was maintained. In total, approximately 20 mL of 5 mM MβCD:cholesterol (8:1) was applied per experiment. At the end of the incubation, MβCD:cholesterol was thoroughly washed out with 10 mL of extracellular solution to ensure complete separation of MβCD-containing solution from capsaicin. Finally, capsaicin was applied a second time, at the same concentration used earlier. In one set of experiments, capsaicin concentration was 1 μM; in the second set of experiments, capsaicin concentration was 10 nM.

The effect of cholesterol enrichment on capsaicin-induced currents was analyzed as follows: In each experiment, *I*_max_ was calculated by subtracting the basal current from the maximal current induced by the first capsaicin application. Similarly, the value *I* was obtained by subtracting the current level after cholesterol washout from the maximal current induced by the second capsaicin application. The ratio *I*/*I*_max_ represents the relative change in capsaicin-induced currents following cholesterol incubation. Histograms represent mean ± standard error of the mean (SEM), and statistical significance was determined using paired two-tailed Student’s *t*-tests.

A separate set of whole-cell experiments was performed in T-REx™-293 cells transiently expressing rTRPV1^G563S^. Currents were evoked by capsaicin (1 μM), followed by a thorough washout with 20 mL extracellular solution. Subsequently, 5 mM MβCD:cholesterol (8:1) solution was applied. For statistical analysis of rTRPV1 ^G563S^ experiments, *I*_max_ was defined in each experiment as the peak current evoked by capsaicin application without subtraction of basal currents, because in several experiments the basal current represented a substantial fraction of *I*_max_. Current levels were measured at the end of capsaicin washout and following cholesterol incubation and normalized to *I*_max._ Thus, the ratio *I*/*I*_max_ represents the relative change in rTRPV1^G563S^ capsaicin-induced currents following washout or cholesterol enrichment, respectively. Histograms represent SEM with *n* = 7, and statistical significance was determined using paired two-tailed Student’s *t*-tests.

### Docking analysis

Docking analysis was performed on the entire transmembrane region of rTRPV1 (PDB ID: 3J9J), using AutoDock Vina [12]. Predicted cholesterol-rTPRV1 poses were superimposed and clustered if they had a Root Mean Square Deviation (RMSD) of <2 Å. However, due to subtle differences in the cholesterol tail orientation among the poses, the clustering criterion was subsequently adjusted to <8 Å. Each cluster was ranked according to its binding energy, and the top 10 clusters were analyzed to identify interacting residues within 4.5 Å of the cholesterol molecule.

## Results and discussion

Membrane cholesterol is known to modulate the activity of numerous ion channels, either through direct interactions with channel proteins or indirectly by altering their association with other regulatory components. In most cases, cholesterol binding inhibits channel activity, and putative cholesterol-binding sites have been identified in several ion channels, typically located within nonannular hydrophobic pockets between transmembrane helices [13]. Docking analysis based on the cryo-EM structure of rat TRPV1 revealed a putative cholesterol-binding pocket [13] (Figure 1). Consistent with the binding of vanilloids near this region [4,5], cholesterol was predicted to bind with a partial overlap to a capsaicin molecule [6]. Therefore, we hypothesized that the colocalization of cholesterol and capsaicin molecules within the vanilloid-binding pocket may lead to cholesterol inhibition of capsaicin activation of the TRPV1 channel.

To test this hypothesis, we examined the effects of membrane cholesterol enrichment on capsaicin-induced TRPV1 activity in T-REx™-293 cells expressing the channel. We did not examine the effect of cholesterol sequestration because it has no effect on capsaicin-induced TRPV1 current in HEK cells [7]. Membrane cholesterol levels were increased by using cholesterol-loaded methyl-β-cyclodextrin (MβCD). MβCD is a cyclic oligosaccharide with a hydrophilic exterior and a hydrophobic central cavity that binds cholesterol with high affinity. In its cholesterol-loaded form, MβCD efficiently delivers cholesterol to the plasma membrane, while it can be rapidly removed by washing [7]. It has been reported that MβCD directly sequester capsaicin [14]. Consequently, MβCD and capsaicin should not be co-applied in functional assays. In our experiments, we thoroughly washed MβCD containing solutions thus preventing co-application of capsaicin and MβCD.

**Figure 2.**
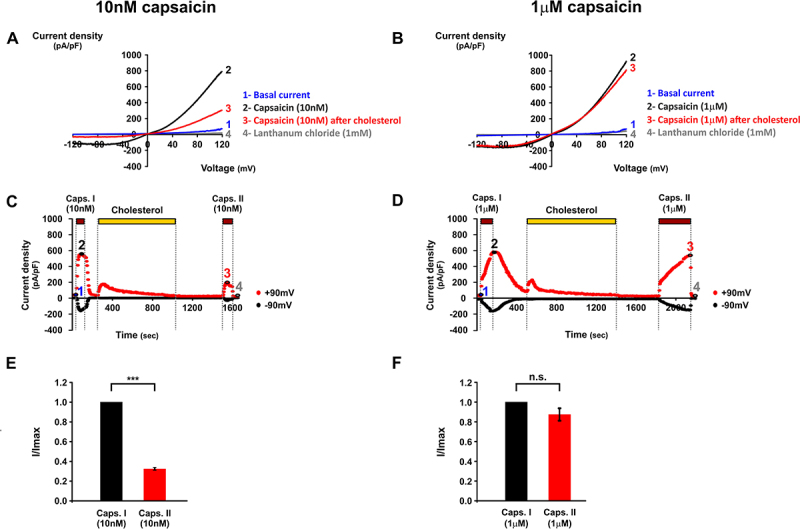
Cholesterol enrichment inhibits rTRPV1^WT^ activity induced by 10 nM capsaicin but not by 1 μM capsaicin. Whole-cell recordings were performed using voltage ramps from −150 mV to +150 mV.

To directly examine the effect of cholesterol enrichment on capsaicin-induced TRPV1 function, whole-cell experiments were performed with T-REx™-293 cells transiently expressing rTRPV1, using voltage ramps (see Materials and Methods section). A 10-min incubation with MβCD:cholesterol significantly reduced capsaicin-induced TRPV1 currents, but only when a low capsaicin level (10 nM) was used. When a near saturating, 100-folds higher capsaicin level (1 μM) was applied, no significant reduction in the current amplitude was observed (Figure 2(C–F)). In each experiment, capsaicin was first applied to establish a baseline response (control), followed by a bath washout prior to MβCD:cholesterol application. Perfusion with MβCD:cholesterol was maintained for 10 min, a time that is sufficient to increase the membrane cholesterol level by at least 40% [15]. After this incubation period, MβCD:cholesterol solution was thoroughly washed out to remove any residual MβCD, and capsaicin was reapplied at the same concentration. At 10 nM capsaicin, the second response was markedly smaller than the initial control response obtained prior to cholesterol treatment (Figure 2(C,E)), whereas at 1 μM capsaicin, the two responses were similar in amplitude (Figure 2(D,F)). For quantification, the maximal current amplitude of the first capsaicin response was defined as *I*_max_ and the maximal current of the second response was expressed relative to this value (Figure 2(E)–F). These results suggest that elevated membrane cholesterol inhibits capsaicin-induced TRPV1 activity depending on capsaicin levels, as reflected by the reduced current amplitude in the second capsaicin response in experiments using low, but not high, capsaicin levels.

A–B. Representative I/V curves obtained with 10 nM (A) or 1 μM (B) capsaicin. Basal currents (1, blue), capsaicin-induced currents (2, black), capsaicin-induced currents after cholesterol treatment (3, red), and lanthanum chloride (1 mM)-inhibited currents (4, gray) are shown. C–D. Time courses of representative experiments corresponding to panels A and B. Each point represents current at ±90 mV. E-F. Comparison of mean current amplitudes before (Caps I, black) and after (Caps II, red) MβCD:cholesterol incubation, measured at +90 mV. For 10 nM capsaicin (E), cholesterol enrichment significantly reduced currents to 32 ± 3.4% of Imax (n = 8, mean ± S.E.M.; p ≤ 0.001, two-tailed paired t-test). For 1 μM capsaicin (F), MβCD:cholesterol incubation had no significant effect (n = 8, mean ± S.E.M.; p >0.05).

**Figure 3.**
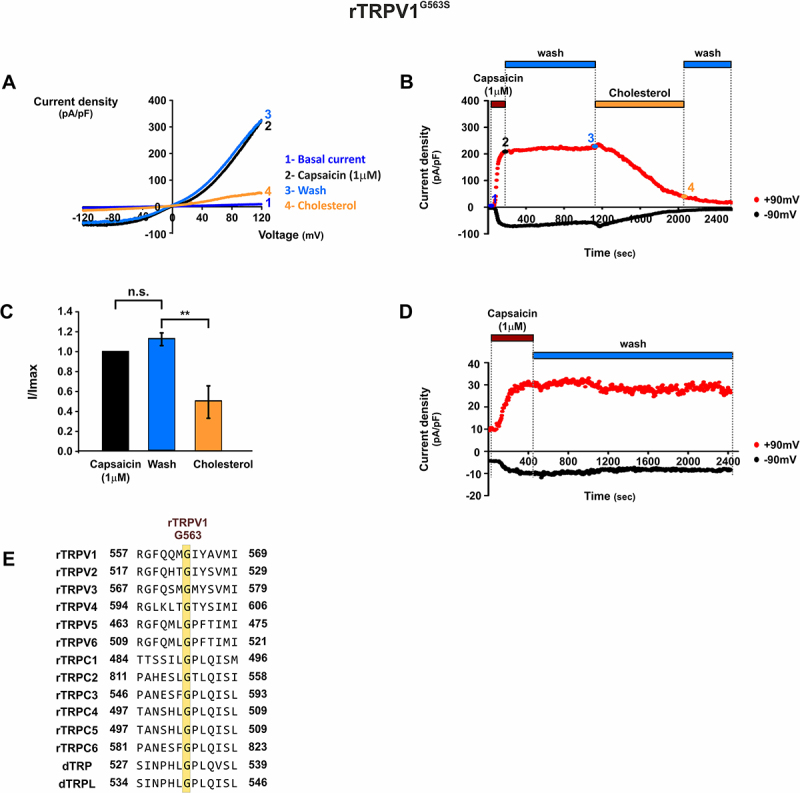
Effect of cholesterol enrichment on rTRPV1^G563S^ mutant channel activity. Whole-cell recordings were performed using voltage ramps from −150 mV to +150 mV.

Docking analysis identified two potential cholesterol-binding pockets in TRPV1. The first is a cleft between the S1–S2 helices at the protein–membrane interface (see also [16]), and the second is a pocket formed between two subunits, where the S5–S6 helices of one subunit interact with S4 and S4–S5 linker of an adjacent subunit (Figure 1). A similar binding pocket of vanilloids was previously identified by cryo-EM [4,5]. Based on these structural studies, we focused on this interunit region as a putative site of cholesterol interaction. The functional interplay between cholesterol and capsaicin was further evaluated using site-directed mutagenesis of Gly563, a conserved residue in the S4–S5 linker at the vanilloid-binding pocket (Figure 3(E)). Our docking analysis identified this residue within the predicted cholesterol-binding region, making it of particular interest given its conservation across all TRPV and TRPC family members (Figure 3(E)) [17]. Previous studies in mice expressing the homologous mTRPV1^G564S^ mutant have demonstrated impaired inflammatory thermal pain responses, reduced capsaicin sensitivity, and diminished histamine-induced itch [18]. Similarly, heterologous expression of rTRPV1^G563S^ in HEK cells resulted in increased basal currents, reduced capsaicin sensitivity, slower activation kinetics, and incomplete current deactivation [19]. To examine the effect of cholesterol enrichment on rTRPV1^G563S^ function, T-REx™-293 cells transiently expressing the mutant channel were subjected to whole-cell recordings. rTRPV1^G563S^ expressing cells revealed reduced sensitivity to capsaicin. Therefore, measurements were carried out using 1 μM instead of 10 nm capsaicin (Figure 3(A–D)), followed by perfusion with MβCD:cholesterol solution, after washout of the agonist. Interestingly, rTRPV1^G563S^-expressing cells displayed both reduced capsaicin sensitivity and persistent current during washout of the agonist (Figure 3(A–C)). Strikingly, this persistent TRPV1-dependent current was suppressed by subsequent perfusion with MβCD:cholesterol (Figure 3(A–C)) supporting the inhibitory role of cholesterol on capsaicin-induced currents. Because Gly563 was predicted to reside within the putative cholesterol-binding pocket, these results strongly suggest a co-localization of the vanilloid-binding pocket with a cholesterol binding pocket within the TRPV1 channel.

A. Representative *I*/*V* curves of *rTRPV1*^*G563S*^-dependent currents. Basal currents (1, blue), capsaicin-induced currents (2, black), capsaicin-induced currents after capsaicin washout (3, light blue), and currents after cholesterol treatment (4, yellow) are shown.

B. Time course of the representative experiment in A, showing currents at ±90 mV. Capsaicin (1 μM, dark red) was applied and then washed out (light blue). MβCD:cholesterol (yellow) was perfused and incubated for 10 min, followed by washout (light blue). Capsaicin application produced sustained currents that did not deactivate after washout, whereas MβCD:cholesterol perfusion markedly reduced current amplitude. Numbers represent the time-points from which corresponding traces were taken in A.

C. Mean current values after capsaicin washout (light blue) and after MβCD:cholesterol application (yellow), normalized to the initial I_max_ (black). Cholesterol enrichment significantly reduced currents to 50 ± 16% of *I*_max_ (*n* = 6, mean ± SEM; *p* ≤ 0.001, two-tailed paired *t*-test).

D. Representative time course showing prolonged capsaicin washout (~30 min). The decrease in current (4, A, B) corresponds directly to cholesterol enrichment rather than spontaneous channel rundown.

E. G563 in rTRPV1 is conserved across TRPV and TRPC channels. The sequence between positions 557–569 in rTRPV1 was aligned with rTRPC1, rTRPC2, rTRPC4, and rTRPC5. The corresponding sequence in rTRPC1 (amino acids 484–496) was aligned with rTRPC3, rTRPC6, *Drosophila* TRP, and *Drosophila* TRPL. All alignments were performed using BLASTP.

Previous studies have already identified a resident lipid, likely a phosphatidylinositol species [5], occupying the capsaicin-binding position in the apo state of TRPV1 [4], which is engaged in polar interactions with the S4–S5 linker via its inositol headgroup [4,5]. However, the cryo-EM structure of human TRPV1 in complex with SAF312 [3] together with docking analysis [6,13] and our functional data suggest that cholesterol occupies a very similar localization within the vanilloid-binding pocket as the putative phosphatidylinositol species proposed in these earlier studies. Collectively, the data of the present study support a mechanism in which capsaicin and cholesterol compete within the vanilloid-binding pocket of TRPV1, with cholesterol binding stabilizing the closed channel conformation, while its replacement by capsaicin enables channel activation.

## Data Availability

PDB ID: 3J9J (https://doi.org/10.2210/pdb3J9J/pdb).

## References

[1] Luu DD, Owens AM, Mebrat MD, et al. A molecular perspective on identifying TRPV1 thermosensitive regions and disentangling polymodal activation. Temp (Austin). 2023;10(1):67–8. doi: 10.1080/23328940.2021.1983354 PMC10177694 37187836

[2] Saha S, Ghosh A, Tiwari N, et al. Preferential selection of arginine at the lipid-water-interface of TRPV1 during vertebrate evolution correlates with its snorkeling behaviour and cholesterol interaction. Sci Rep. 2017;7(1):16808. doi: 10.1038/s41598-017-16780-w 29196683 PMC5711878

[3] Fan J, Ke H, Lei J, et al. Structural basis of TRPV1 inhibition by SAF312 and cholesterol. Nat Commun. 2024;15(1):6689. doi: 10.1038/s41467-024-51085-3 39107321 PMC11303535

[4] Elokely K, Velisetty P, Delemotte L, et al. Understanding TRPV1 activation by ligands: insights from the binding modes of capsaicin and resiniferatoxin. Proc Natl Acad Sci U S A. 2016;113(2):E137–145. doi: 10.1073/pnas.1517288113 26719417 PMC4720335

[5] Zhang K, Julius D, Cheng Y. Structural snapshots of TRPV1 reveal mechanism of polymodal functionality. Cell. 2021;184(20):5138–5150.e5112. doi: 10.1016/j.cell.2021.08.012 34496225 PMC8488022

[6] Brandwine-Shemmer T, Minke B, Levitan I. Inhibition of TRPV1 by an antagonist in clinical trials is dependent on cholesterol binding. Cell Calcium. 2024;124:102957. doi: 10.1016/j.ceca.2024.102957 39357317

[7] Picazo-Juárez G, Romero-Suárez S, Nieto-Posadas A, et al. Identification of a binding motif in the S5 helix that confers cholesterol sensitivity to the TRPV1 ion channel. J Biol Chem. 2011;286(28):24966–24976. doi: 10.1074/jbc.M111.237537 21555515 PMC3137070

[8] Hanson SM, Newstead S, Swartz KJ, et al. Capsaicin interaction with TRPV1 channels in a lipid bilayer: molecular dynamics simulation. Biophys J. 2015;108(6):1425–1434. doi: 10.1016/j.bpj.2015.02.013 25809255 PMC4375533

[9] Mohanta S, Das NK, Saha S, et al. Capsaicin-insensitivity of TRPV1-R575D mutant located at the lipid-water-interface region can be rescued by either extracellular Ca. Neurochem Int. 2024;179:105826. 39117000 10.1016/j.neuint.2024.105826

[10] Ohtani Y, Irie T, Uekama K, et al. Differential effects of alpha-, beta- and gamma-cyclodextrins on human erythrocytes. Eur J Biochem. 1989;186(1–2):17–22. doi: 10.1111/j.1432-1033.1989.tb15171.x 2598927

[11] Christian AE, Haynes MP, Phillips MC, et al. Use of cyclodextrins for manipulating cellular cholesterol content. J Lipid Res. 1997;38(11):2264–2272. doi: 10.1016/S0022-2275(20)34940-3 9392424

[12] Trott O, Olson AJ. Autodock vina: improving the speed and accuracy of docking with a new scoring function, efficient optimization, and multithreading. J Comput Chem. 2010;31(2):455–461. doi: 10.1002/jcc.21334 19499576 PMC3041641

[13] Barbera NA, Minke B, Levitan I. Comparative docking analysis of cholesterol analogs to ion channels to discriminate between stereospecific binding vs. stereospecific response, Channels. (Austin). 2019;13(1):136–146. doi: 10.1080/19336950.2019.1606670 31033379 PMC6527060

[14] Abdelnabi H, Alshaer W, Azzam H, et al. Loading of capsaicin-in-cyclodextrin inclusion complexes into PEGylated liposomes and the inhibitory effect on IL-8 production by MDA-MB-231 and A549 cancer cell lines. Z Naturforsch C J Biosci. 2021;76(11–12):503–514. doi: 10.1515/znc-2021-0018 34036759

[15] Lee SY, Choi HK, Kim ST, et al. Cholesterol inhibits M-type K+ channels via protein kinase C-dependent phosphorylation in sympathetic neurons. J Biol Chem. 2010;285(14):10939–10950. doi: 10.1074/jbc.M109.048868 20123983 PMC2856299

[16] Espinoza-Arcos LG, González-Avendaño M, Zuñiga-Bustos M, et al. Exploring a peripheral PIP2-binding site and its role in the alternative regulation of the TRP channel superfamily. J Gen Physiol. 2025;157(6). doi: 10.1085/jgp.202413574 40844491

[17] Boukalova S, Marsakova L, Teisinger J, et al. Conserved residues within the putative S4-S5 region serve distinct functions among thermosensitive vanilloid transient receptor potential (TRPV) channels. J Biol Chem. 2010;285(53):41455–41462. doi: 10.1074/jbc.M110.145466 21044960 PMC3009871

[18] Duo L, Hu L, Tian N, et al. TRPV1 gain-of-function mutation impairs pain and itch sensations in mice. Mol Pain. 2018;14:1744806918762031. doi: 10.1177/1744806918762031 29424270 PMC5846932

[19] Boukalova S, Touska F, Marsakova L, et al. Gain-of-function mutations in the transient receptor potential channels TRPV1 and TRPA1: how painful? Physiol Res. 2014;63:S205–213. doi: 10.33549/physiolres.932658 24564660

